# Antiasthmatic Effects of Sanglong Pingchuan Decoction through Inducing a Balanced Th1/Th2 Immune Response

**DOI:** 10.1155/2018/2629565

**Published:** 2018-06-11

**Authors:** Binnian Zhu, Jun Dong, Xiangyun Gao, Yanfei He, Hongxiang Sun

**Affiliations:** ^1^Key Laboratory of Animal Virology of Ministry of Agriculture, College of Animal Sciences, Zhejiang University, Hangzhou 310058, China; ^2^Shaoxing Hospital of Traditional Chinese Medicine, Shaoxing 312000, China

## Abstract

**Objective:**

To investigate the antiasthmatic effects of Sanglong pingchuan decoction (SLPCD) and to explore its mechanisms of action.

**Methods:**

The serum, bronchoalveolar lavage fluid (BALF), and lung tissues from OVA-induced allergic asthma mice were collected 24 h after the last administration. Lung pathological changes were observed by H&E staining. The inflammatory cells in BALF were counted by flow cytometry. The levels of total IgE in serum and cytokines in BALF were determined by ELISA. The expression levels of cytokine mRNA in lung were assayed by qRT-PCR.

**Results:**

SLPCD significantly inhibited airway inflammation, reduced inflammatory cells in BALF, reduced the levels of total IgE in serum and Th2 cytokines (IL-10 and IL-13) in BALF, and downregulated the mRNA expression levels of Th2 cytokines (IL-4, IL-5, IL-10, and IL-13) in lung of asthmatic mice. However, SLPCD remarkably elevated the level of Th1 cytokine IFN-*γ* in BALF and upregulated the mRNA expression levels of Th1 cytokines (IL-2 and IFN-*γ*) in lung of asthmatic mice.

**Conclusion:**

SLPCD could attenuate airway inflammation and alleviate the pathogenesis in asthma mice through inducing a balanced Th1/Th2 response and could act as an effective drug for treatment of asthma.

## 1. Introduction

Asthma is a complex, chronic respiratory disease that is characterized by bronchial hyperresponsiveness, airway inflammation and remodeling, airflow obstruction, and infiltration of different kinds of inflammatory cells induced by cytokines and other mediators [1, 2]. Asthma is caused by environmental factors such as allergens, viruses, and occupational exposures in genetically predisposed individuals; however, its definitive cause is unclear [3]. The incidence of asthma has significantly increased worldwide, especially in young children, to become one of the most common respiratory diseases [4–6]. It is estimated that 300 million individuals are affected by asthma among different countries [7].

Currently, corticosteroids are the most effective drugs for the treatment of asthma [8]. Although these drugs can improve asthma symptoms, they do not cure this disease [9]. These agents also have serious side effects, particularly in children, including immune suppression, secondary infections, muscle atrophy, osteopenia, osteoporosis, cataract, and glaucoma [10, 11]. Therefore, the continued search for new, safe, and effective drugs is of utmost importance.

Many traditional Chinese medicines have been used in treating asthma for centuries and are still widely used in Asian countries [12]. Recently, the mechanism of action of numerous herbal formulas used for treating allergic asthma have been reported [13, 14]. Various plant-derived natural products with anti-inflammatory properties have also been screened for potential application in the treatment of asthma [15–17].

Sanglong pingchuan decoction (SLPCD) is a hospital preparation prescribed by Professor Qing Chang, a national veteran doctor of traditional Chinese medicine inherited studio mentor, in Shaoxing Hospital of Traditional Chinese Medicine. SLPCD is derived from Shegan Mahuang Tang by addition and subtraction. Shegan Mahuang Tang is a well-known traditional Chinese prescription medicine first mentioned in Jin Kui Yao Lve. As the representative formulation of dissipate cold, relieving exterior syndrome, warming lung, eliminating phlegm, and relieving asthma, Shegan Mahuang Tang is widely used for the treatment of asthma, bronchitis in children, bronchial asthma, pneumonia, the elderly acute and chronic bronchitis, emphysema, and allergic rhinitis [18, 19]. The formula of SLPCD consisted of thirteen traditional Chinese medicines listed in Table 1. A randomized trial demonstrated that SLPCD is effective in the treatment of asthma, especially in reducing the exacerbation rate. However, the efficacy of SLPCD has not been proven by controlled experiments.

In the present study, to provide experimental basis for the clinical application of SLPCD in treating asthma and to explore its mechanisms of action, using OVA-induced allergic asthma mouse model, the anti-inflammatory effects of SLPCD were investigated by histological examination, enumeration of immune cells in bronchoalveolar lavage fluid (BALF), quantization of total IgE in serum, cytokine in BALF, and the mRNA expression of cytokine in lung tissues.

## 2. Materials and Methods

### 2.1. Plant Materials and Reagents

Cortex Mori (batch number: 140901), Pheretima Aspergillum (batch number: 150406), honey-processed Herba Ephedrae (batch number: 150302), Rhizoma Belamcandae (batch number: 141213), Semen Armeniacae Amarum (batch number: 150312), stir-baked Fructus Perillae (batch number: 150115), Scorpio (batch number: 150126), Semen Descurainiae (batch number: 150208), honey-fried Flos Farfarae (batch number: 150301), Herba Houttyniae (batch number: 150327), Rhizoma Fagopyri Dibotrydis (batch number: 150308), Radix Angelicae Sinensis (batch number: 150213), and Radix et Rhizoma Glycyrrhizae (batch number: 140917) were purchased From Shaoxing Hospital of Chinese Medicine and were authenticated by Professor Yonghai Jiang from the Shaoxing Institute for Food and Drug Control, Zhejiang, China. The standards of chlorogenic acid and rutin were purchased from Zhejiang Institute for Food and Drug Control, Hangzhou, China, and the purities of both compounds were more than 98%.

Ovalbumin (OVA) was purchased from Sigma-Aldrich Chemical Co., St. Louis, MO, USA; mouse IL-10, IL-13, and IFN-*γ* detecting ELISA kits were purchased from Wuhan Boster Biological Technology Co. Ltd., Hubei, China; mouse IgE ELISA kit was from RayBiotech Inc., Norcross, GA, USA, fetal calf serum (FCS) was from Gibco, Grand Island, NY, USA. The purified anti-mouse CD16/CD32 (Fc Receptor block), Ly-6G–FITC (clone: RB6-8C5), F4/80 antigen PE-Cy5 (clone: BM8), Ly-6C-APC (clone HK1.4), Fc epsilon Receptor 1 alpha (FceR1)–APC (clone: MAR-1), CD117 (c-Kit)-PE-Cy5 (c-Kit, clone: 2B8), and siglec-F-PE (clone: E50-2440) antibodies were purchased from eBioscience, Inc., San Diego, CA, USA. Trizol reagent was purchased from Invitrogen, Carlsbad, CA, USA; revert Aid™ M-MLV reverse transcriptase was from Fermentas, Amherst, NY, USA; ribonuclease inhibitor and oligo(dT)_18_ were from Sangon Biotech (Shanghai) Co., Ltd., China; FastStart Universal SYBR Green Master (ROX) was from Roche Diagnostics, Indianapolis, IN, USA. Aluminum hydroxide gel (Alum) was purchased from China Animal Husbandry Industry Co., Ltd., Beijing, China; dexamethasone (Dex) was from Hubei Tianyao Pharmaceutical Co., Ltd., Xiangyang, China.

### 2.2. Preparation of SLPCD

Prescription medicines of SLPCD (440 g) were macerated in 8 aliquots of distilled water for 30 min and then boiled water for 1 h. After the first decoction, dregs were boiled in 6 aliquots of distilled water for another 30 min. The supernatant obtained from the twice decoction was filtered through a sterile gauze and concentrated by a rotavapor (Buchi, Switzerland) under reduced pressure at 65°C to a final concentration of 1.1 g crude drug/ml. The concentrated decoction was stored at −20°C and subsequently diluted by distilled water to several different concentrations before use.

### 2.3. High Performance Liquid Chromatography (HPLC) Analysis of SLPCD

HPLC analysis was performed using a Diamon C18 column (250 mm × 5 mm, 5 *μ*m) and Waters 2996 PDA detector on the Water 600E HPLC instrument. The mobile phase was a mixture of acetonitrile (A) and 0.1% phosphoric acid (B). A linear gradient elution was conducted as follows: 0–8 min at 2% A, 8–35 min at 6% A, 35-40 min at 8% A, 40-45 min at 13% A, 45–70 min at 14% A, 70–80 min at 17% A, and 80–90 min at 17–2% A. The flow rate was 1 ml/min and the injection volume was 10 *μ*l. Column temperature was constantly kept at 30°C. The HPLC analysis of standards (chlorogenic acid and rutin) and the samples were carried out under the established experimental condition as mentioned above.

### 2.4. Experimental Animals

Female BALB/c mice aged 5 weeks were purchased from Shanghai Experimental Animal Center of Chinese Academy of Sciences, Shanghai, China (certificate no. SCXK 2007-0005). Mice were acclimatized for 1 week prior to use. Rodent laboratory chow and tap water were provided* ad libitum* and maintained under controlled conditions with a temperature of 24 ± 1°C, humidity of 50 ± 10%, and a 12/12-h light/dark cycle. All the procedures were in strict accordance with the PR China legislation on the use and care of laboratory animals and with the guidelines established by Institute for Experimental Animals of Zhejiang University and were approved by the university committee for animal experiments.

### 2.5. Sensitization, Challenge, and Treatment of Mice

**Figure 1:**
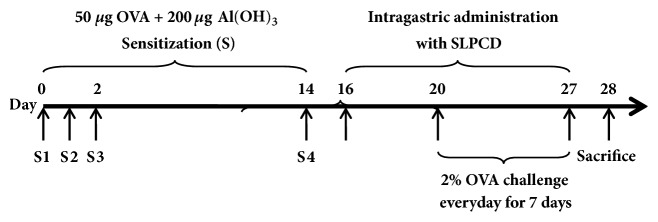
Experimental protocol for OVA-induced asthmatic model and treatment processes.

Female BALB/c mice were divided into six groups: normal control group (NC), model control group (MC), positive control group (treated with Dex, 2 mg/kg), and SLPCD groups (5.5, 11, and 22 g/kg). Each group consisted of ten mice. The mouse airway inflammation was induced by OVA. Animals were immunized by intraperitoneal injection of a solution containing 50 *μ*g of OVA and 200 *μ*g of aluminum hydroxide for 3 days. A boosting sensitization was given on day 14. One week after the last sensitization, mice were exposed to aerosolized 2% OVA using PARI TurboBOY N (1205) nebulizer (PARI GmbH, German) for 1 h per day for 8 days. Four days before being challenged, the sensitized mice were orally administered with SLPCD at the doses of 5.5, 11, and 22 g/kg or intraperitoneally injected with 2 mg/kg of dexamethasone (Dex) every day for 12 days. Model control groups received the same volume of saline. The dose volume was 0.2 ml/10g body weight. Ten mice as the normal control group were only sensitized and challenged with saline. The procedures employed for OVA sensitization and challenges, as well as the treatment with SLPCD, were shown in Figure 1.

### 2.6. Lung Histopathology

The lung tissues were collected 24 h after the last administration and then fixed with 4% paraformaldehyde, embedded in paraffin, and sectioned at 5 *μ*m thicknesses. A series of microsections was stained with haematoxylin and eosin (H&E) for histological assessment.

### 2.7. Bronchoalveolar Lavage Fluid (BALF) Collection and Flow Cytometry

BALF was collected by lavage of the lung with phosphate buffered solution (PBS) delivered through an endotracheal catheter 24 h after the last administration. BALF was centrifuged for 5 min and the cells were washed with ice-cold PBS containing 2% FCS. Cells were blocked with 0.5 *μ*g of purified anti-mouse CD16/CD32 (FcR block) antibody for 10 min on ice to inhibit nonspecific staining and then stained with the combination of anti-mouse Ly-6G–FITC, F4/80 antigen–PE-Cy5 and Ly-6C-APC, or FceR1–APC, CD117–FITC and Siglec-F–PE antibodies at the room temperature for 30 min in the dark. The stained cells were washed with ice-cold PBS and resuspended in PBS. One hundred thousand viable cells per treatment were analyzed using a BD FACScan flow cytometer using CellQuest software.

### 2.8. Measurement of the Total IgE Antibody in Serum

The serum was collected 24 h after the last administration. The total IgE antibody was detected in individual serum samples by the RayBio® mouse IgE ELISA kit. The sera samples diluted 1:1250 or IgE standard were added to 96-well plates coated with specific anti-mouse IgE antibody. The plates were incubated for 2.5 h at room temperature and then washed four times. The detecting antibody was added to each well. Plates were incubated at room temperature for 1 h before addition of horseradish peroxidase-conjugated ABC. After incubation for 45 min, plates were washed and developed with TMB for 30 min at room temperature. The reaction was stopped by addition of 20 *μ*l of stop solution. The absorbance was measured on ELISA reader (BIO-RAD 680) at 450 nm.

### 2.9. Measurement of Cytokines in BALF

The concentrations of Th2 (IL-4, IL-5, IL-10, and IL-13) and Th1 (IFN-*γ* and IL-2) cytokines in BALF were detected using commercial ELISA kits as previously [20].

### 2.10. Quantitative Real-Time PCR (qRT-PCR)

The mRNA expression levels of Th2 (IL-4, IL-5, IL-10, and IL-13) and Th1 (IFN-*γ* and IL-2) cytokines in lung tissues were determined using qRT-PCR. The total RNA was isolated from homogenized lung tissues using the Trizol reagent according to the manufacture's protocol, and reverse transcription was performed as previously [21]. Then amplification was carried out in 20 *μ*l reaction using FastStart Universal SYBR Green Master. Real-time PCR was performed using an ABI 7400 Real-Time PCR System. Primers for qRT-PCR were synthesized by Sangon Biotech (Shanghai) Co., Ltd. (China), and the sequences were listed in Table 2. The qPCR cycling was performed as follows: initial denaturation at 95°C for 10 min followed by 40 cycles of denaturation at 95°C for 10 s, annealing at 60°C for 1 min. GAPDH was used as an endogenous control. Primer amplification efficiency and specificity were verified for each set of primers. The expression levels of the tested genes relative to GAPDH were determined using the 2^ΔΔCt^ method and as fold induction [22].

### 2.11. Statistical Analysis

The data were expressed as mean ± standard deviation (SD) and examined for their statistical significance of difference with ANOVA and a Tukey post hoc test. *P*-values of less than 0.05 were considered to be statistically significant.

## 3. Results

### 3.1. HPLC Profile of SLPCD

**Figure 2:**
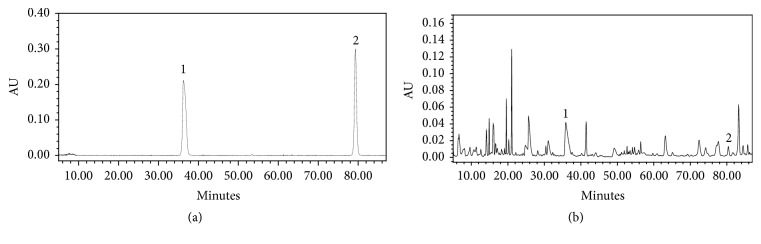
HPLC chromatograms of SLPCD. Representative chromatograms of standard reference compounds (a) and SLPCD (b) were shown. ① Chlorogenic acid and ② rutin.

The contents of chlorogenic acid and rutin in SLPCD were analyzed using HPLC. As compared with standard reference compounds, chlorogenic acid and rutin were identified and determined (Figure 2). The regression equations from the calibration curve of standards were A = 3719.7C – 102166 (R^2^=0.9999) and A = 9137.3C – 408038 (R^2^=0.9996) for chlorogenic acid and rutin, respectively. The concentrations of chlorogenic acid and rutin in SLPCD were 1.4 mg/g and 0.15 mg/g, respectively.

### 3.2. SLPCD Attenuated Airway Inflammation in Asthmatic Mice

**Figure 3:**
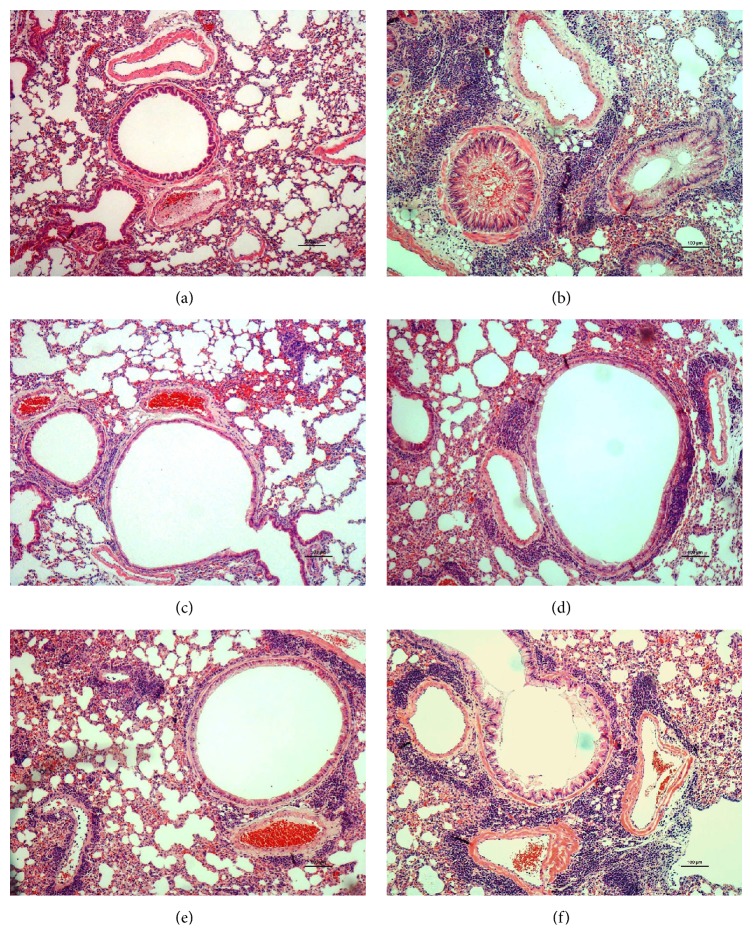
Effects of SLPCD treatment on histopathological changes in the lungs. The lung sections were stained using hematoxylin and eosin. The light photomicrographs shown were representative of lung sections from ten mice per group. (a) Normal control (NC); (b) model control (MC); (c) dexamethasone (Dex, positive control); (d) SLPCD (5.5 g/kg); (e) SLPCD (11 g/kg); and (f) SLPCD (11 g/kg).

The effect of SLPCD on lung inflammation in OVA-induced asthmatic mice was observed* via* H&E staining, and the results were shown in Figure 3. The asthma model mice exhibited more serious interstitial, peribronchiolar, and perivascular inflammatory cell infiltration in the lung compared with normal control mice. As a positive control, Dex significantly relieved the infiltration of lung interstitial, peribronchiolar, and perivascular inflammatory cells in asthma mice. The inflammatory infiltrates in the lungs of the OVA-challenged mice were also significantly attenuated by SLPCD compared with the model control.

### 3.3. SLPCD Reduced Inflammatory Cells in BALF of Asthmatic Mice

**Figure 4:**
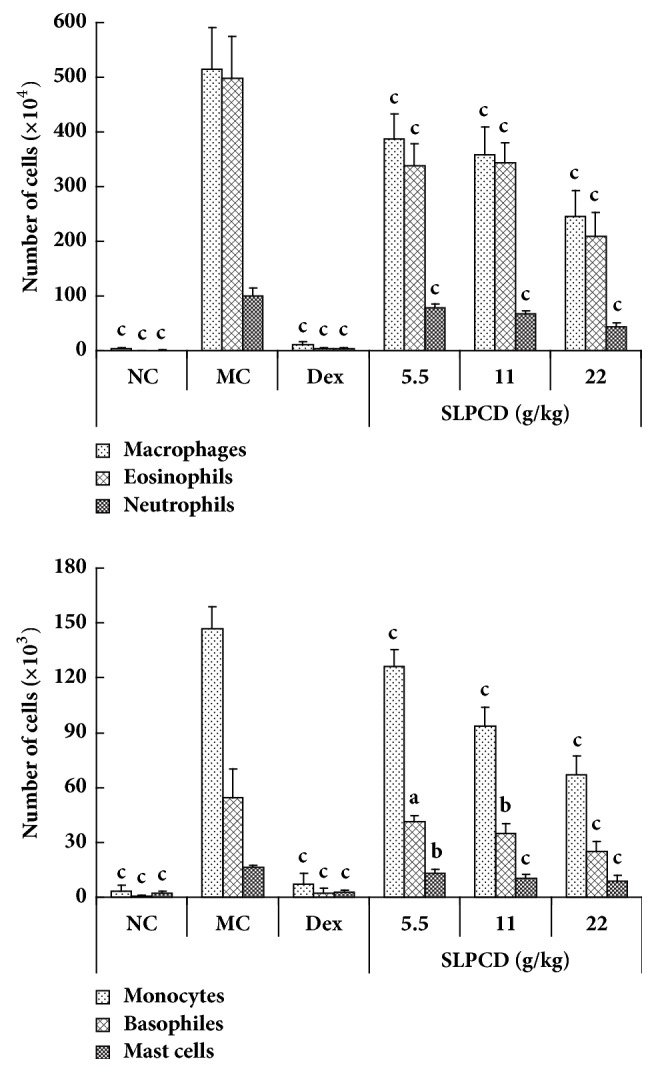
Effect of SLPCD on the recruitment of inflammatory cells into BALF in OVA-induced asthma mice. The BALF was collected 24 h after the last administration and cell composition was analyzed by FACS described in the text. The values are presented as means ± SD (*n* = 10). Significant differences compared to asthmatic model control group (MC) are designated as ^a^*P* < 0.05, ^b^*P* < 0.01, and ^c^*P* < 0.001. Dex: dexamethasone (positive control), NC: normal control group.

To ascertain the effects of SLPCD on the infiltration of inflammatory cells into the lung of OVA-induced asthmatic mice, the count of the inflammatory cells including eosinophils (Sigilec F^+^), basophiles (CD117^+^), neutrophils (Ly-6C^+^y-6G^High^), macrophages (F4/80^high^), monocytes (Ly6G^−^Ly6C^+^), and mast cells (FcER1^+^) in BALF was performed by flow cytometry. As shown in Figure 4, the numbers of eosinophils (1756-fold), macrophages (114-fold), neutrophils (110-fold), basophiles (91.7-fold), monocytes (45.8-fold), and mast cells (6.9-fold) in the BALF from asthmatic mice were markedly elevated compared with normal control mice. Dex significantly decreased the numbers of a variety of inflammatory cells tested in the BALF of asthmatic mice (*P* < 0.001), almost close to the levels of normal mice. The numbers of these inflammatory cells in BALF from asthmatic mice were significantly dose-dependently reduced by SLPCD compared with the model control group (*P* < 0.05, P < 0.01, or *P* < 0.001).

### 3.4. SLPCD Decreased Serum Total IgE Levels of Asthmatic Mice

**Figure 5:**
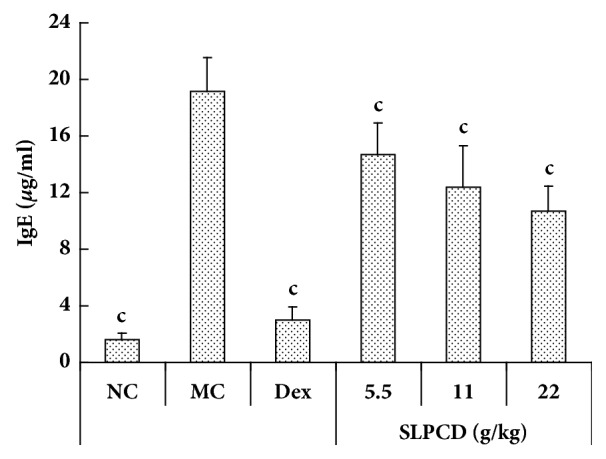
Effects of SLPCD on the levels of total IgE in serum of OVA-induced asthma mice. Serum was collected 24 h after the last administration and the levels of total IgE were measured using ELISA kits described in the text. The values are presented as means ± SD (*n* = 10). Significant differences compared to asthmatic model control group (MC) are designated as ^c^*P* < 0.001. Dex: dexamethasone (positive control) and NC: normal control group.

The effects of SLPCD on the serum total IgE levels in asthmatic mice were shown in Figure 5. The serum total IgE antibody levels in asthmatic mice were significantly higher than those in normal control mice (*P* < 0.001). As a positive drug, Dex significantly decreased the serum total IgE antibody levels in OVA-induced asthmatic mice (*P* < 0.001). The serum total IgE antibody levels in the asthmatic mice were significantly dose-dependently decreased by SLPCD compared with model control group (*P* < 0.001).

### 3.5. SLPCD Modulated the Levels of Cytokines in BALF of Asthmatic Mice

**Figure 6:**
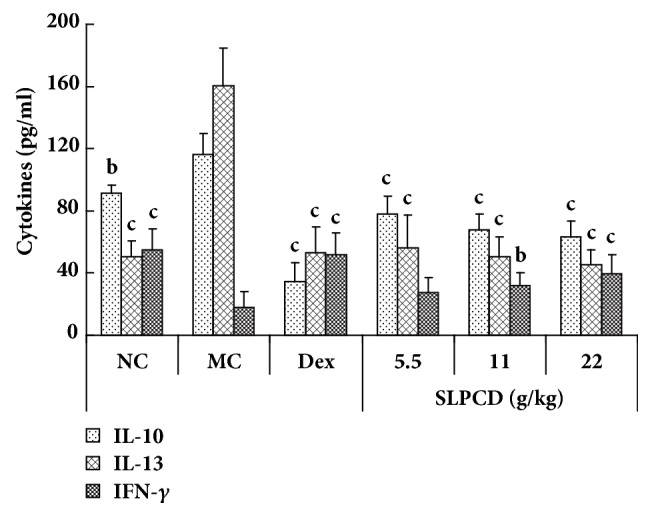
Effects of SLPCD on the levels of cytokines in BALF in OVA-induced asthma mice. BALF was collected 24 h after the last administration. The levels of Th1 (IL-10 and IL-13) and Th1 (IFN-*γ*) cytokines in BALF were measured using ELISA kits described in the text. The values are presented as means ± SD (*n* = 10). Significant differences compared to asthmatic model control group (MC) are designated as ^b^*P* < 0.01 and ^c^*P* < 0.001. Dex: dexamethasone (positive control) and NC: normal control group.

The levels of Th2 (IL-4, IL-5, IL-10, and IL-13) and Th1 (IFN-*γ* and IL-2) cytokines in BALF were measured by ELISA. As shown in Figure 6, OVA challenge led to the significant increase in the level of IL-10 and IL-13 and decrease in that of IFN-*γ* in the BALF of the asthmatic mice compared with the normal control group (*P* < 0.01 or *P* < 0.001). However, the contents of IL-4, IL-5, and IL-2 in BALF of model control mice were found to be very low and close to detection limit. The administration of SLPCD at three doses and Dex significantly decreased the levels of IL-13 and IL-10, as well as remarkably elevated that of IFN-*γ* in BALF of OVA-induced asthmatic mice as compared with model control mice (*P* < 0.01 or *P* < 0.001) (Figure 6).

### 3.6. SLPCD Regulated the mRNA Expression Levels of Cytokines in Lung Tissues of Asthmatic Mice

The effects of SLPCD on the mRNA expression of Th1 and Th2-type cytokines in lung tissues of OVA-induced asthmatic mice were detected using qRT-PCR, and the results were shown in Table 3. As compared with the normal control mice, the expression levels of Th2 cytokine mRNAs (IL-4, IL-5, IL-10, and IL-13) were markedly upregulated and those of Th1 cytokine mRNAs (IL-2 and IFN-*γ*) were significantly downregulated in lung tissues of asthmatic mice (*P* < 0.001). The administration of SLPCD at three doses and Dex resulted in significant downregulation of the mRNA expression levels of Th2 cytokines IL-4, IL-5, IL-10, and IL-13 and upregulation of those of Th1 cytokines IL-2 and IFN-*γ* in lung tissues from asthmatic mice compared with the asthmatic model groups (*P < 0.05, P* < 0.01, or* P* < 0.001).

## 4. Discussion

The asthma has become a public health problem worldwide, especially in developed countries [23]. Glucocorticoids are the first-line therapy for the treatment and control of asthma. Although these drugs could improve asthma symptoms, they were strictly controlled for persistent use due to their serious side effects [11]. Thus, new asthmatic therapeutic approaches are necessary. Many traditional Chinese medicines exhibit various biological activities, including antibacterial, antifungal, anti-inflammatory, antianaphylaxis, and immunomodulatory effects, which have potential for asthma treatment. Various models of allergic bronchopulmonary inflammation in experimental mice have been described. OVA-induced allergic asthma mouse model replicates many features of human asthma, which has gained extensive acceptance [24]. Therefore, we investigated the antiasthmatic effects of SLPCD and explored its molecular mechanisms using this model.

SLPCD consists of 13 traditional Chinese medicines. Cortex Mori, Pheretima Aspergillum, and Herba Ephedrae are the primary component that disperse the lung and relieve asthma. Semen Armeniacae Amarum, stir-baked Fructus Perillae, Semen Descurainiae, and honey-fried Flos Farfarae are ancillary components that reduce phlegm, relieve cough, and asthma. The adjuvant components include Rhizoma Belamcandae, Herba Houttyniae, and Rhizoma Fagopyri Dibotrydis that clear away the heat evil and expel superficial evils, as well as Scorpio and Radix Angelicae Sinensis that disperse blood stasis, dredge collateral, spasmolyze and stop asthma. Radix et Rhizoma Glycyrrhizae harmonizes all drugs. Taken together, all components complement each other to achieve the outcomes of dispersing the lung, descending Qi, and relieving cough and asthma. The quality control of SLPCD used in this study was undertaken using HPLC, and two reference chemicals (chlorogenic acid and rutin) were identified as major index components (Figure 2).

Allergic asthma is defined as an airway acute-on-chronic inflammatory disease with characteristic eosinophilic recruitment, hyperplasia of goblet cells, mucus hypersecretion, collagen deposition, smooth muscle cell hypertrophy, and subepithelial fibrosis [25, 26]. In this study, the effects of SLPCD treatment on lung inflammation in OVA-challenged asthmatic mice were assessed* via* H&E staining. The typical allergic accumulation and infiltration of inflammatory cells were observed in lung tissues of asthmatic model mice. The treatment of SLPCD reduced the inflammatory infiltrates in the lung tissues of asthmatic mice (Figure 3), suggesting that SLPCD markedly alleviated airway inflammation.

The inflammatory immune cells play a key role in the pathogenesis and severity of asthma [27]. The inflammatory cells recruited from regional lymph nodes to the airways could secrete cytokines and chemokines, resulting in airway hyperreactivity [28]. Reducing the inflammatory cells in the airway is an effective way to treat asthma [29–32]. To confirm that SLPCD can inhibit inflammatory cell infiltration, we further counted eosinophils, macrophages, neutrophils, basophiles, monocytes, and mast cells in BALF. OVA inhalation led to significant increases in the counts of these six sorts of inflammatory cells in the BALF (Figure 4). The increase folds were 1756, 114, 110, 91.7, 45.8, and 6.9 folds for eosinophils, macrophages, neutrophils, basophiles, monocytes, and mast cells, respectively, indicating a satisfactory establishment of an exacerbated asthma model in this study. Treatment with SLPCD significantly and dose-dependently diminished the number of these inflammatory cells, especially eosinophils, in BALF in comparison with asthmatic mice (Figure 4). These results suggested that SLPCD reduced the infiltration of the inflammatory cell into the lung in OVA-induced asthmatic mice, being consistent with the results of the histopathological observation.

It is well known that the elevation of IgE antibody levels was correlated with allergic inflammation of the airways and bronchial hyperreactivity in asthma model mice [33]. Coyle et al. [34] reported that anti-IgE antibodies attenuated eosinophilic airway inflammation and bronchial hyperreactivity in asthmatic mice. Allergen-induced IgE triggered eosinophils, basophils, and mast cells to secrete cytokines IL-4, IL-5, IL-10, and IL-13 [35]. Therefore, we assessed serum total IgE levels and found that SLPCD could significantly lower the serum total IgE levels in OVA-challenged asthmatic mice (Figure 5).

The imbalance of Th1/Th2 cells is considered to be the immunological cause of asthma [36, 37]. In asthma, the activity of Th1 cells is reduced, while Th2 cell activity is activated, resulting in an increase in Th2 type cytokines and a decrease in Th1 cytokines [38–40]. Th2 cells migrated to the lung secrete the prototypical Th2 type cytokines IL-4, IL-5, and IL-13, resulting in goblet cell hyperplasia, bronchoconstriction, and eosinophilia in the airway [41–43]. In vitro, IL-13 can mediate the production of IgE and may play a key role in the pathogenesis of IgE-mediated allergic diseases [44]. IL-13 can also enhance survival of eosinophils and contribute to the pathological activities of these cells in asthma [45]. Mice overexpressing IL-13 replicate several features of asthma, including pulmonary eosinophilia, epithelial hyperplasia, airways obstruction, and hyperresponse to cholinergic.

Suppressing the production of Th2 cytokines would prove to be useful for allergen immunotherapy [46]. On the other hand, Th1-activated cells can inhibit the inflammation of asthmatic airways [47]. Th1 cytokines, such as IFN-*γ*, inhibit allergen-induced eosinophil recruitment and IgE release* via* downregulating GATA-3, IL-4, and IL-5 expression in the lung tissues of asthma model [3, 48]. In this study, we estimated the effect of SLPCD on the levels of cytokines in BALF from the asthmatic mice. The results showed that SLPCD dose-dependently not only reduced the levels of IL-13 and IL-10, but also elevated the level of IFN-*γ* in the BALF of the asthmatic mice (Figure 6). The inhibitory action of SLPCD treatment on production of Th2 cytokines was consistent with its effect on the levels of total IgE in serum.

It was reported that IL-5 plays an important role in the proliferation, differentiation, maturation, and migration to tissue sites of eosinophils [49]. The expression levels of IL-5 mRNA in bronchial biopsies from asthmatics were upregulated compared with nonasthmatic controls [50]. The mRNA expression levels of IL-5 were correlated with the clinical severity of asthma [51]. The accumulative allergen provocation upregulated IL-4 mRNA expression in BALF cells and peripheral CD4^+^ and CD8^+^ T cells [52]. IL-10 have also been indicated to participate in asthma [53]. The upregulation of IL-13 transcripts in BAL cells of patients with mild asthma after low-dose allergen challenged is consistent with its higher expression level of IL-13 mRNA in bronchial mucosa [54]. The effects of SLPCD treatment on the mRNA expression level of cytokines in lung tissues from the asthmatic mice were further determined using qRT-PCR. SLPCD treatment significantly downregulated the mRNA expression levels of Th2 cytokines (IL-4, IL-5, IL-10, and IL-13) and upregulated those of Th1 cytokines (IL-2 and IFN-*γ*) in lung from asthmatic mice. These results further confirmed the regulatory effects of SLPCD treatment on Th1 and Th2 cytokine responses in lung tissues of asthmatic mice.

In conclusion, SLPCD could significantly inhibit airway inflammation, reduce inflammatory cells in BALF, decrease the serum total IgE levels, and modulate the contents of cytokines in BALF and the mRNA expression of cytokines in lung from asthmatic mice. These results suggested that SLPCD could attenuate airway inflammation and alleviate the pathogenesis in a mouse model of asthma through inducing a balanced Th1/Th2 response and validate its clinical use as an effective drug in the treatment of asthma.

## Figures and Tables

**Table 1:** Composition of SLPCD.

Chinese name	Pharmaceutical name	Botanical or zoological name	Family and part used	Amount (g)
Sang Bai Pi	Mori Cortex	*Morus alba* L.	Moraceae, root bark	30
Di Long	Pheretima	*Pheretima aspergillum* (E. Perrier)	Megascolecidae, body	18
Ma Huang	Ephedrae Herba	*Ephedra sinica *Stapf	Ephedraceae, aerial part	10
Ku Xing Ren	Armeniacae Semen Amarum	*Prunus armeniaca* L. var. *ansu* Maxim.	Rosaceae, seed	10
Zi Su Zi	Perillae Fructus	*Perillae frutescens *(L.) Britt.	Labiatae, seed	15
Ting Li Zi	Descurainiae Semen	*Descurainia Sophia* (L.) Webb. Ex Prantl.	Cruciferae, seed	15
Kuan Dong Hua	Farfarae Flos	*Tussilago farfara* L.	Compositae, bud	18
She Gan	*Belamcandae Rhizoma*	*Belamcanda chinensis *(L ) DC.	Iridaceae, rhizome	10
Yu Xing Cao	Houttuyniae Herba	*Houttuynia cordata* Thunb.	Saururaceae, aerial part	30
Jin Qiao Mai	Fagopyri Dibotrydis Rhizoma	*Fagopyrum dibotrys* (D. Don) Hara	Polygonaceae, rhizome	30
Quan Xie	Scorpio	*Buthus martensii *Karsch	Buthidae, body	6
Dang Gui	Angelicae Sinensis Radix	*Angelica sinensis (*Oliv.) Diels	Umbelliferae, root	18
Gan Cao	Glycyrrhizae Radix et Rhizoma	*Glycyrrhiza uralensis *Fisch.	Leguminosae, root and rhizome	10

The plant name has been checked with http://www.theplantlist.org mentioning the data of accessing that website.

**Table 2:** Primer sequences used for qRT-PCR.

Gene	Primer sequence	Product size (bp)
GAPDH	5′-AGCCTCGTCCCGTAGACAA-3′	104
	5′-AATCTCCACTTTGCCACTGC-3′	
IL-2	5′-CCCAAGCAGGCCACAGAATTGAAA-3′	81
	5′-AGTCAAATCCAGAACATGCCGCAG-3′	
IFN-*γ*	5′- TCTTGAAAGACAATCAGGCCATCA -3′	233
	5′- GAATCAGCAGCGACTCCTTTTCC -3′	
IL-4	5′-CAAACGTCCTCACAGCAACG-3′	203
	5′-CTTGGACTCATTCATGGTGC-3′	
IL-5	5′- GCTGGCCTCAAACTGGTAATGTA -3′	100
	5′- GGCAATGGTGCATGTCTGTAACCTC -3′	
IL-10	5′- GCTCTTACTGACTGGCATGAG -3′	105
	5′- CGCAGCTCTAGGAGCATGTG -3′	
IL-13	5′- GCTTGCCTTGGTGGTCTCGCC -3′	175
	5′- GGGCTACACAGAACCCGCCA -3′	

**Table 3:** Effect of SLPCD on the mRNA expression levels of cytokines in lung tissues of OVA-induced asthmatic mice.

Group	Dose	IL-4	IL-5	IL-10	IL-13	IL-2	IFN-*γ*
NC	-	1.00±0.14^c^	1.00±0.23^c^	1.00±0.19^c^	1.00±0.31^c^	1.00±0.19^c^	1.00±0.13^c^
MC	-	38.91±2.96	5.22±0.64	7.49±0.23	142.3±14.6	0.73±0.13	0.59±0.15
CTX (mg/kg)	50	19.08±2.62^c^	2.56±0.42^c^	5.07±0.67^c^	44.00±7.74^c^	1.20±0.20^c^	2.14±0.34^c^
SLPCD (g/kg)	5.5	21.70±4.64^c^	4.06±0.38^b^	6.68±0.68^a^	71.26±10.62^c^	1.28±0.20^c^	1.12±0.20^c^
	11	21.30±3.71^c^	3.74±0.37^c^	6.21±0.44^c^	65.4±11.99^c^	1.33±0.26^c^	1.53±0.21^c^
	22	23.09±3.27^c^	3.07±0.34^c^	6.03±0.65^c^	61.51±8.77^c^	1.49±0.34^c^	1.71±0.23^c^

The lung tissues were collected 24 h after the last administration and the mRNA expression levels of GAPDH and cytokines were detected by qRT-PCR using specific primers. The housekeeping gene GAPDH was used as endogenous control. The values are presented as means ± SD (*n* = 10). Significant differences compared to asthmatic model control group (MC) are designated as ^a^*P* < 0.05, ^b^*P* < 0.01, and ^c^*P* < 0.001. Dex: dexamethasone (positive control) and NC: normal control group.
